# FPC4: a new cytoskeletal component in *T.brucei*

**DOI:** 10.1186/2046-2530-4-S1-P45

**Published:** 2015-07-13

**Authors:** A Albisetti, C Florimond, A Sahin, M Eggenspieler, O Cingal, D Robinson, M Bonhivers

**Affiliations:** 1 MFP, CNRS UMR5234, Bordeaux, France

## 

*Trypanosoma brucei *is the causative agent of African sleeping sickness. It possesses a single flagellum, which additional to its mobility role, is an important sensory and signaling organelle [1,2]. The flagellum exits the cell from a membrane invagination, called the flagellar pocket (FP), region where endo- and exocytosis occur. A ring-shaped structure, called the flagellar pocket collar (FPC), encloses the FP defining the flagellum exit point. BILBO1 is the first FPC protein identified and is essential for the FPC and FP biogenesis [2]. A *T. brucei *gDNA yeast two-hybrid (Y2H) screen identified several BILBO1 protein partners, including FPC4 a protein we are characterizing at the molecular and functional level.

Our anti-FPC4 antibody and endogenous FPC4-myc cell confirmed that FPC4 localizes both on, and close to the FPC. RNAi down-regulation of FPC4 has no impact on cell proliferation. However, over-expression of FPC4 leads to filament formation and to a mild, but reduced growth defect. Co-immunolocalization demonstrated that BILBO1 relocalizes to the FP4 filaments indicating BILBO1-FPC4 interaction and the role of FPC4 in FPC structure. Y2H shows that BILBO1 interacts with the C-terminal domain of FPC4 and we are currently identifying the minimal interaction domains, with the long-term objective of a drug screen to block this interaction. Expression of FPC4 in a heterologous system (human cells) suggests that the N-terminal domain binds to microtubules (MT). This was confirmed by *in vitro *MT binding assays and is ongoing. We are also characterizing FPC4 function in bloodstream form.

Our preliminary data suggest that FPC4 could be a FPC-microtubule linker involved in FPC segregation during the cell cycle.
